# Supplementary material for: A combined risk model shows viability for personalized breast cancer risk assessment in the Indonesian population: A case/control study
Source: PLoS One. 2025 May 15;20(5):e0321545. doi: 10.1371/journal.pone.0321545 (PMC12080871; doi:10.1371/journal.pone.0321545)
Supplement: S1 File — This questionnaire was administered to study participants after obtaining their informed consent. (PDF) [file pone.0321545.s001.pdf]

Order Form

Specimen Details		Lab Barcode Accession No.
Specimen Type*	:............................	
Ordered by*	:............................	
Consultant	:............................	
Clinic's Name*	:............................	
Clinic's Address*	:............................	
Collection Date*	:............................	

Patient Details

Full Name* :.. **ID (KTP)*** :..

Date of Birth* (DD-MM-YYYY) :.. **Phone Number*** : (+62)

Age* :............................ **Height*** :……………………cm **Weight*** :………………………kg

Gender* ☐ Female ☐ Male **Menopausal Status*** : ☐ Pre-menopause ☐ Post-menopause*

Address* :
.... **Postal Code*** :............................

Ethnicity*

☐ Ashkenazi Jew	☐ Filipino	If selecting "Indonesian", please specify further (e.g., Maluku, Javanese, Sundanese, etc.)
☐ Caucasian	☐ Hawaiian	
☐ African	☐ Indonesian	
☐ Hispanic (US Born)	☐ Other Pacific Islander	
☐ Hispanic (Foreign Born)	☐ Other Asian	
☐ Chinese	☐ Unknown	
☐ Japanese		

General Detail

Eating / drinking before sample collection:_________________minute / hour (cross out one)

Food / drink before sample collection: ____

**Assessment Related to Examination (Breast Cancer Risk)**		
Age of first menstrual period	☐ Unknown<br>☐ 7-11 years old	☐ 12-13 years old<br>☐ > 13 years old
Age at first live birth	☐ Unknown<br>☐ No births<br>☐ < 20 years old	☐ 20-24 years old<br>☐ 25-29 years old<br>☐ >= 30 years old
Numbers of first degree relative with history of breast cancer (Child, Parent, or Siblings)	☐ Unknown<br>☐ 0	☐ 1<br>☐ >1
History of breast biopsy with a benign diagnosis (Fibroadenoma Mammae)<br>)**If you have never undergone a biopsy, select 'Unknown'. If you have undergone biopsy and malignancy or cancer was found, select '0'.	☐ Unknown<br>☐ 0	☐ 1<br>☐ >1
History of atypical hyperplasia)**<br>)**If you have never undergone a biopsy, select 'Unknown'. Accumulation of abnormal cells in the breast / Pre-cancer. If you have never had a breast biopsy, please fill in the 'Unknown' box.	☐ Yes<br>☐ No	☐ Unknown
**Relevant Clinical Information (Diagnosis and Medical History)**		
Patient has had prior thoracic radiotherapy before age 30	☐ Yes	☐ No
Patient has had a history of LCIS)**<br>)**Lobular carcinoma in situ (LCIS) is an area (or areas) of abnormal cell growth that increases a person's risk of developing invasive breast cancer later on in life.	☐ Yes	☐ No
Patient has had a history DCIS)**<br>)**Ductal carcinoma in situ (DCIS) is an area of abnormal cells inside a milk duct in the breast. DCIS is noninvasive, meaning it hasn't spread out of the milk duct and has a low risk of becoming invasive.	☐ Yes	☐ No
Patient has had a previous diagnosis of breast cancer.	☐ Yes)*	☐ No

Order Form

)* **If Yes:**<br>Age of the diagnosis:<br>Estrogen receptor status (If data exists):<br>Progesterone receptor status (If data exists):<br>HER2 receptor status (If data exists):			
Patient has had a second diagnosis of breast cancer		☐ Yes	☐ No
Patient has had a previous diagnosis of triple-negative breast cancer (ER-negative, PR-negative, HER2-negative)		☐ Yes	☐ No
**Lifestyle**			
How many servings of fruit and vegetables the patient eats a day?	☐ 1 serving	☐ 2 – 3 servings	☐ Once a week or less
How often does the patient exercise per week?	☐ 150 minutes<br>☐ 100 minutes	☐ 60 minutes<br>☐ 30 minutes	☐ Unknown
Does the patient smoke?	☐ Yes	☐ No	
How often does the patient drink alcohol?	☐ 1-2 drinks a day	☐ > 3 drinks per day	☐ Rarely / doesn't drink alcohol
Use of oral contraceptive	☐ Never	☐ Former	☐ Currently using
Use of hormone replacement therapy	☐ Never<br>☐ Former (any type)	☐ Currently using Estrogen only<br>☐ Currently using other type	
**Preference**			
Preference for risk-reducing therapy		☐ Yes	☐ No
Preference for lifestyle modification		☐ Yes	☐ No
Preference for risk-reducing surgery		☐ Yes	☐ No
Preference for risk-reducing for genetic counseling for multi-gene panel		☐ Yes	☐ No
**Genetic Predisposition to Breast Cancer (BRCA1/2, p53, PTEN, or other gene mutations)**			
Did the patient have any genetic predisposition to breast cancer?		☐ Unknown	☐ Yes ☐ No
How many of the patient's family relatives have known genetic predisposition to breast cancer?		☐ Unknown<br>☐ __________(Please fill 0 - 10)<br>☐ >10	
**Screening and Monitoring (Part 1)**			
When was the last time the patient had breast cancer screening?	☐ Never<br>☐ In the last 3 months	☐ In the last 6 months<br>☐ 1 year ago	☐ 2 year ago<br>☐ > 2 year ago
When was the last time the patient had ovarian cancer screening?	☐ Never<br>☐ In the last 3 months	☐ In the last 6 months<br>☐ 1 year ago	☐ 2 years ago<br>☐ > 2 years ago
**Screening and Monitoring (Part 2)**			
Patient had normal results for breast cancer screening according to clinical practice baseline assessment.	☐ Unknown	☐ Yes	☐ No
Patient had normal results from gynecologic assessment to assess the risk of endometrial cancer.	☐ Unknown	☐ Yes	☐ No
**Test Ordered**			
**Deliver To :**<br>[illegible]	**Notes**		

"I have filled out this survey truthfully and to the best of my ability. If the information provided is found to be inaccurate, I understand that it may impact the accuracy and quality of my test results."
